# Clustering of cancer among families of cases with Hodgkin Lymphoma (HL), Multiple Myeloma (MM), Non-Hodgkin's Lymphoma (NHL), Soft Tissue Sarcoma (STS) and control subjects

**DOI:** 10.1186/1471-2407-9-70

**Published:** 2009-02-27

**Authors:** Helen H McDuffie, Punam Pahwa, Chandima P Karunanayake, John J Spinelli, James A Dosman

**Affiliations:** 1 Canadian Centre for Health and Safety in Agriculture, University of Saskatchewan, Royal University Hospital, 103 Hospital Drive, Saskatoon, SK S7N 0W8, Canada; 2 Cancer Control Research, British Columbia Cancer Agency, 2-111, 675 West 10th Avenue, Vancouver, British Columbia V5Z 1L3, Canada; 3 Department of Community Health & Epidemiology, University of Saskatchewan, Health Science Building, 107, Wiggins Road, University of Saskatchewan, Saskatoon, Saskatchewan S7N 5E5, Canada

## Abstract

**Background:**

A positive family history of chronic diseases including cancer can be used as an index of genetic and shared environmental influences. The tumours studied have several putative risk factors in common including occupational exposure to certain pesticides and a positive family history of cancer.

**Methods:**

We conducted population-based studies of Hodgkin lymphoma (HL), Multiple Myeloma (MM), non-Hodgkin's Lymphoma (NHL), and Soft Tissue Sarcoma (STS) among male incident case and control subjects in six Canadian provinces. The postal questionnaire was used to collect personal demographic data, a medical history, a lifetime occupational history, smoking pattern, and the information on family history of cancer. The family history of cancer was restricted to first degree relatives and included relationship to the index subjects and the types of tumours diagnosed among relatives. The information was collected on 1528 cases (HL (n = 316), MM (n = 342), NHL (n = 513), STS (n = 357)) and 1506 age ± 2 years and province of residence matched control subjects. Conditional logistic regression analyses adjusted for the matching variables were conducted.

**Results:**

We found that most families were cancer free, and a minority included two or more affected relatives. HL [(OR_adj _(95% CI) **1.79 (1.33, 2.42)]**, MM **(1.38(1.07, 1.78))**, NHL **(1.43 (1.15, 1.77)**), and STS cases **(1.30(1.00, 1.68)) **had higher incidence of cancer if any first degree relative was affected with cancer compared to control families. Constructing mutually exclusive categories combining "family history of cancer" (yes, no) and "pesticide exposure ≥10 hours per year" (yes, no) indicated that a positive family history was important for HL **(2.25(1.61, 3.15))**, and for the combination of the two exposures increased risk for MM **(1.69(1.14,2.51))**. Also, a positive family history of cancer both with **(1.72 (1.21, 2.45)) **and without pesticide exposure **(1.43(1.12, 1.83)) **increased risk of NHL.

**Conclusion:**

HL, MM, NHL, and STS cases had higher incidence of cancer if any first degree relative affected with cancer compared to control families. A positive family history of cancer and/or shared environmental exposure to agricultural chemicals play an important role in the development of cancer.

## Background

At the cellular level, all cancers are genetic [1]. A positive family history of chronic diseases including cancer can be used as an index of genetic and shared environmental influences [2,3]. The four types of tumours under investigation (Hodgkin Lymphoma, Multiple Myeloma, Non-Hodgkin's Lymphoma and Soft Tissue Sarcoma) have several putative risk factors [4-63] in common, which include occupational exposure to certain chemicals and specific pesticides [4-37], a positive family history of cancer specifically of the hematopoietic system [29,30,37-54], a positive personal history of cancer or other diseases, as well as certain types of medical treatments for cancer and personal habits [38,55-63].

Researchers have stated that more than 80% of human cancers can be attributed to environmental factors [2,3,64,65]. Inherited and shared environmental influences among family members (such as tobacco smoke, viruses, agricultural chemicals and dusts, animal contact) that contribute to cancer's development can sometimes be delineated by (a) deconstructing the familial pattern of tumour occurrences (parent-offspring versus sibling-sibling), and (b) collecting detailed information of historical exposure by utilizing various methods. Simultaneously, in order to compare the family history of cancer, we conducted population-based case-control studies of Hodgkin Lymphoma, Multiple Myeloma, Non-Hodgkin's Lymphoma and Soft Tissue Sarcoma among male incident case and control subjects in six Canadian provinces using postal questionnaires in order to compare details of family history of cancer among them.

## Methods

The detailed methodology has been previously published [38]. Briefly, we contacted male incident cases aged 19 years or older with a first diagnosis of Hodgkin Lymphoma (ICD-9 201), Multiple Myeloma (ICD-9 203), Non-Hodgkin's Lymphoma (ICD-9 200 or 202), Soft Tissue Sarcoma (ICD-9 code 171 and any other ICD-9 category with certain specified morphology codes), who were diagnosed on or after September 1^st^, 1991 until at the latest on or before December 31^st^, 1994, and who were residents of six Canadian provinces (Quebec, Ontario, Manitoba, Saskatchewan, Alberta, and British Columbia). The Cases were ascertained from provincial cancer registries except in Quebec where hospital registries were used. The control subjects were males aged 19 years and older selected from provincial Health Insurance records (Alberta, Saskatchewan, Manitoba, and Quebec), telephone listings (Ontario) or Voter's lists (British Columbia). The random control subject selection was stratified by age ± 2 years to be comparable to the age distribution of the entire case group within each province.

The family history section of the postal questionnaire used in this study was developed and validated by McDuffie *et al *[66]. The family history section was designed to collect information on the number and sex of siblings and children of the index subjects. Additional queries ascertained information on the index subject's children who were either stillborn, born with a genetic disease and/or a birth defect. The definition of family was restricted to first degree relatives; parents, siblings and children. Within each family there were three generations: parents, sibling and children. Half-siblings, adopted siblings or children and stepchildren were excluded. Both living and deceased first degree relatives were included. The information on family history of cancer included the affected individual's relationship of to the index subject, the site of the cancer, and the year and province of diagnosis. Reported cancer in family members was not verified.

The questionnaire instrument was also used to collect personal demographic data, health history, smoking pattern and lifetime occupational history. Exposure to broad classes of pesticides (herbicides, insecticides, fumigants, fungicides, and algaecides) was included as these were potential confounders of the putative association between a positive family history of cancer and each of the tumours of interest. Pesticide exposure was a calculated variable that required affirmative responses to questions concerning exposure to any combination of the pesticides listed. Cumulative exposure of ten hours per year or more to pesticides was considered a positive response.

### Data Management and Statistical Analysis

The questionnaires were reviewed for completeness and consistency of the responses by the coordinators in each province. The data were computer coded in the province of origin using SPSS-DE [67]; they were also checked and transported on disks to the coordinating centre where the data were reviewed for completeness and internal consistency. The analytic approach was that suggested by Breslow *et al *[68]. We used SAS [69] computer software to conduct the analyses.

We conducted descriptive statistical analyses and compared family history of cancer among each case group to the control group's family history data. In addition four mutually exclusive categories combining the variables family history of cancer (yes, no) and pesticide exposure ≥10 hours/year (yes, no) were constructed. Similar mutually exclusive categories were formed for family history of cancer and smoking status (non-smokers, ex-smoker or current smoker). We conducted conditional logistic regression analyses adjusted for the matching variables age and province of residence. We report adjusted odds ratios (OR_adj_) and 95% confidence intervals (95% CI).

### Ethical Approval

The letters of informed consent, the questionnaires and other written material provided to potential subjects were submitted to and approved by each of the relevant agencies in each province. For Saskatchewan, the University of Saskatchewan Biomedical Research Ethics Board (#89-12); for British Columbia, the University of British Columbia Behavioural Research Ethics Board (#B91-185); for Quebec, the University of McGill Human Ethics Board; for Alberta, the University of Alberta Health Research Ethics Board (Biomedical Panel); for Manitoba, the University of Manitoba Biomedical Research Ethics Board; and for Ontario, the University of Toronto Health Sciences Research Board approved the study protocol. All information that could be used to identify the individuals remained within the province of origin under the control of each province's principal investigator. After receiving permission to contact a potential case by the attending physician, a letter of informed consent and a questionnaire was mailed. Relatives of cases known to be deceased at ascertainment were not contacted because of the need to obtain detailed information concerning occupation and occupational exposures including a variety of types of pesticides. Control subjects were contacted directly by mail. Living control subjects were eligible. Additional methodological details have been previously published [38].

## Results

Information about the family history of cancer in first degree relatives of 1528 cases: (HL (n = 316), MM (n = 342), NHL (n = 513), STS (n = 357)) and 1506 control subjects was obtained. Descriptive characteristics of the families studied are shown in Tables 1 through 4 [See additional file 1: Table 1; additional file 2: Table 2; additional file 3: Table 3; additional file 4: Table 4]. The age distribution, the mean age and standard deviation, the number of first degree relatives, the mean number of siblings and offspring, and the mean number of first degree family members including parents among controls and within each case group are shown in Table 1 [See additional file 1: Table 1]. HL cases were much younger compared to MM, NHL, and STS cases. HL families were the smallest with smaller mean number of siblings, sons and daughters compared to families of other case groups and controls. The four categories: small, medium, large and largest categories were used to define the family size (for definition see footnote under Table 2 [See additional file 2: Table 2]). There were distinctive differences in the family size patterns (chi square = 61.5, df = 12, p < .0001) across the case groups and controls, however the family size patterns were not significantly different between STS cases and controls (Table 2 [See additional file 2: Table 2], chi square = 1.80, df = 3, p > 0.05). HL families were the smallest. Families of HL, MM and STS were significantly more likely to have had one affected generation than controls, while a higher proportion of NHL families reported two or more affected generations (Table 3 [See additional file 3: Table 3]). A small minority of families reported affected members in two or more generations. The distribution of affected family members (chi-square = 59.8, df = 8, p < .0001, the categories were zero, one, ≥ 2 affected per family) and the percentage of families with two or more affected individuals are presented in Table 4 [See additional file 4: Table 4]. The majority of families were cancer free, excluding the index subjects. A minority of families included two or more affected first degree relatives of the index subjects.

Table 5 [See additional file 5: Table 5] compares distribution of positive history of cancer in (i) at least one parent; (ii) at least one sibling; and (iii) at least one child of each case group with controls stratified by age distribution of the index subject. Patterns of cancer prevalence are similar among the index subjects diagnosed with four types of cancer (HL, MM, NHL, and STS). The index subjects in the age groups > 40, ≤49, and > 49, ≤59 had higher proportion of at least one parent affected with cancer compared to controls. The proportion of at least one sibling affected with cancer increases with age of the index subject. The siblings of older index subjects may be comparatively older compared to the siblings of younger index subjects and it is well known that risk of cancer increases with age. In most of the age groups, index subjects have higher proportion of at least one sibling affected with cancer compared to controls. For the younger age groups (≤40, and > 40, ≤49), there was hardly any child affected with cancer, because children of the index subjects in these age groups may be younger and had very low risk of developing cancer. The proportions of at least one child affected with cancer are small in the older age groups and among all cases and controls.

Families ascertained through HL, MM, NHL, and STS cases were compared to control families in Table 6 [See additional file 6: Table 6]. Cases of HL [OR_adj _(95% CI 1.79 (1.33, 2.42)], MM [OR_adj _(95% CI 1.38 (1.07, 1.78)], NHL [OR_adj _(95% CI 1.43 (1.15, 1.77)] and STS [1.30 (1.00, 1.68)] were more likely to have at least one first degree relative diagnosed with cancer compared to controls. Although the crude odds ratios (data not shown) for several variables related to history of cancer among families ascertained through HL cases were statistically non-significant, adjustment for age and province of residence produced statistically significant results due to the strength of the associations within certain strata. Index subjects with at least one affected parent with a diagnosis of cancer had increased risk of being diagnosed with HL [1.57 (1.15, 2.14)]. This pattern did not appear in any of the other case/control comparisons. HL [1.94 (1.12, 3.34)], MM [1.71 (1.22, 2.40)] and NHL [1.68 (1.23, 2.31)] cases were significantly more likely to have at least one affected sibling than controls after adjustment for total number of siblings. The excess of siblings was due to more affected sisters among HL families [1.89 (1.05, 3.38)], MM families [1.66 (1.14, 2.40)] and NHL families [1.52 (1.07, 2.17)]. Few children of index subjects (range 0.6% of HL families to 3.7% of MM families) had been diagnosed with cancer.

Among each case/control family group, tumours of the trachea, bronchus, lung (ICD-9 162) and of the female breast (ICD-9 174) were first or second in frequency (data not shown). The female to male ratio for tumours of the trachea, bronchus, and lung was unexpectedly high; 1.45:1 for NHL relatives. There was also one male breast cancer among NHL relatives. Among families of HL cases, lymphoid leukaemia (ICD-9 204) and Hodgkin lymphoma (ICD-9 201) were third and fourth. Among families of MM cases, colon (ICD-9 153) and prostate (ICD-9 185) tumours were third and fourth while stomach (ICD-9 151) and lymphoid leukaemia occupied these ranks among families of NHL cases. Lymphoid leukaemia and liver cancer (ICD-9 155) tied for third place and rectum, sigmoid junction and anus (ICD-9 154) was fourth in frequency among STS families.

In this study, 54.4% of HL cases; 87.7% of MM cases; 79.7% of NHL cases; and 73.7% of STS cases reported to have at least one child compared to 21.4% controls who reported to have at least one child. The HL families reported a higher frequency of birth defects than the control families although there did not appear to be a clustering of specific types (data is not shown). Cleft palate (n = 2) and pyloric stenosis (n = 2) were the only conditions to occur in more than one family while Turner's syndrome, hypospadia and atrophic testis each occurred in one HL family.

Table 7 [See additional file 7: Table 7] displays certain characteristics of the index subjects stratified by family history of cancer. The variables include pesticide exposure ≥ 10 hours per year as a surrogate for occupational and environmental pesticide exposure, cigarette smoking history (non-smokers, ex-smoker or current smoker) and age at diagnosis. Comparisons of each case group with the controls using mutually exclusive categories combining family history status (positive, negative) with either pesticide exposure ≥ 10 hours/year or lesser exposure indicated that a positive family history (but not pesticide exposure) was important for HL [(2.25 (1.61, 3.15)], that the combination of pesticide exposure and a positive family history of cancer increased risk for MM [1.69 (1.14, 2.51)] and that a positive family history of cancer both with [1.72 (1.21, 2.45)] and without [1.43 (1.12, 1.83)] pesticide exposure increased risk of NHL. A family history of cancer both with [2.34 (1.57, 3.48)] and without [1.68 (1.01, 2.78)] a history of cigarette smoking increased risk of HL. Cigarette smoking [1.46 (1.01, 2.12)] and family history [1.73 (1.07, 2.80)] independently and in combination [1.79 (1.22, 2.63)] increased risk of MM while only the combination of cigarette smoking and a positive family history [1.45 (1.07, 1.96)] influenced risk of NHL. There were no statistically significant results of comparisons of STS cases and controls.

Contrary to the expectation that those with a positive family history of cancer will have experienced genetic and/or environmental factors which might lower the age at diagnosis, HL, MM, and STS cases and the age at recruitment of control subjects with a positive family history were significantly older than those with a negative family history.

## Discussion

Data from postal questionnaires based on responses from 513 NHL cases (67.1% of those contacted); 316 HL cases (68.4% of those contacted); 342 MM cases (58.0% of those contacted); 357 STS cases (60.8% of those contacted); and 1506 controls (48.0% of those contacted) were analyzed. Due to budget constraints, this study was restricted to males. Previous studies [66,70-75] have demonstrated that first degree family members are capable of accuracy in reporting chronic illnesses such as cancer among their immediate family members while accuracy declines when the definition of family is expanded [71,75]. Therefore, the study definition of family was restricted to first degree relatives. We collected information on (a) the number and the sex of siblings and children, (b) the children of index subjects who had a genetic disease, a birth defect or who were stillborn; and (c) relatives, specifically on: the type of cancer, the relationship to the index subjects, and the year and province of diagnosis was collected. This comprehensive documentation permitted us to consider family cancer history as stratified by sex, by generation (parental, sibling, children) and by multiple generations and to incorporate the total number of siblings into the analyses.

Consistent with other studies of similar design [39-41,53,66] with a range of 50% to 65% of cancer free families, we found that the majority of families were cancer-free (excluding the index subjects). Previous studies [37-54,66,70-73,75] have shown that the proportion of cancer free families varies with the type of index cancer, with the manner in which the index subject is ascertained, with the age of the index subject and with the size of the family. We found that HL families had the lowest (5.4%) and MM families the highest (17.6%) percent of families with two or more affected individuals per family compared to 8.3% controls families with two or more affected per family.

An exploration of the generational pattern [2,3,30,49,66] of cancer in affected relatives was useful in discussions related to discriminating between inherited and environmental factors contributing to a positive family history of cancer. In our data and that of others as well, the generational patterns of cancer that emerged are parental only, sibling only and multi-generational. A concentration of tumours in one generation, whether parental or sibling, might relate etiologically to time-dependent environmental exposures. In contrast, sibling clustering could also relate to recessively inherited susceptibility. Additional information on exposure to putative carcinogens, such as tobacco smoke or specific pesticides, during the relevant time periods aids in the assessment of the inherited/environmental/interaction triad. In this study, statistically significant increases in risk of at least one affected sibling of the index subject were demonstrated for HL, MM and NHL compared to control families. Among HL families, there were also statistically significant increases in risk in the parental generation (mother was affected, father was unaffected and vice-versa, with; at least one affected parent). This pattern of parental generation involvement was not apparent for MM, NHL or STS families.

Ottman [76] summarized a variety of models illustrating the potential modes of interaction among genetic and environmental factors. By constructing mutually exclusive categories that utilize the variables family history of cancer in combination with either exposure to pesticides > 10 hours per year or cigarette smoking, an examination of the independent and combined effect of a personal characteristic and environmental exposures to known or suspected carcinogenic risk factors for these four types of tumours was examined. Among HL index subjects, those with a positive family history of cancer were at higher risk when unexposed to pesticides while smoking status did not substantially influence the increased risk of HL conferred by a positive family history. These patterns were consistent with the genetic susceptibility etiological hypothesis [1,2] related to HL. In the stratified analysis, the combination of a positive family history and exposure to pesticides increased risk of MM while neither variable did so independently. In a cohort study [77], ex-smokers experienced a three-fold risk of MM compared to life-time non-smokers, with a dose-response effect. Positive histories of cigarette smoking and family history independently and in combination increased risk of MM without an apparent dose effect in this study. This pattern suggests a lack of synergism between the lifestyle factor, cigarette smoking and the inherent factor, positive family history of cancer. A positive family history of cancer both with and without pesticide exposure increased risk of NHL suggesting that a positive family history was more important than pesticide exposure as defined in these analyses. Zahm *et al *[30] reported that refining the definitions of family histories from "any affected family member" to "affected first degree relative with hemapoietic cancer", and pesticide exposure (i.e. from residence on a farm to personal application of a specific chemical class of insecticide) resulted in a dose response pattern among women. The relationship of cigarette smoking to risk of NHL is controversial and smoking risk may only be related to specific subtypes of NHL. In this study, only the combination of cigarette smoking and positive family history significantly influenced risk of NHL, although the odds ratios and 95% confidence intervals for the combinations of positive family history with non smokers and ever smokers were similar. Linet *et al *[78] reported that only the combination of positive family history and cigarette smoking increased risk of NHL.

The major limitation of this study is the fact that the family history of cancer was obtained by report from the index subjects and these reports were not validated by reference to the appropriate provincial cancer registries. There are difficulties involved in validation of self-reported family history of cancer by index subjects as reported by other authors [66,79].

Our study has the advantages of recruiting incident cases and controls from population-based sources covering a large geographical area of Canada. There were three to five controls for each case which increased the statistical power of the study. The use of a common control group and identical methodology permitted comparisons of the importance of a family history of cancer among the four types of cancer studied.

If a familial aggregation of cancer is a surrogate for an inherited, genetic etiology, certain predictions apply [2,66] and we have evaluated our data with respect to several of these. The predictions include: 1. a younger age at diagnosis among those with a positive family history of cancer compared to the general population or those with a negative family history. Our data demonstrated a significantly older age at diagnosis among those with a positive family history for each cancer group and the controls compared to those with a negative family history. Previously, Schneider *et al *[80] demonstrated familial clustering of cancer among relatives of randomly selected cancer cases without regard to age of the index subject. 2. A multigenerational pattern of tumours occurring in both sexes is of interest because the inherited, genetic hypothesis predicts that at least one of the precancerous steps occurred prenatally and each cell within the body therefore has a heightened susceptibility. The predicted pattern was strongest for HL families followed by NHL and MM families. The predicted pattern was not found among STS families. 3. The potential role of environmental factors is complex as specific exposures may act as tumour initiators, or as agents influencing progression or promotion of tumour growth. In the stratified analyses exploring the contributions of pesticide exposure or cigarette smoking independently and in combination with a positive family history of cancer, we did not find strong evidence of synergism in those with combined exposure.

## Conclusion

HL, MM, NHL, and STS cases had higher incidence of cancer if any first degree relative affected with cancer compared to control families. A positive family history of cancer and/or shared environmental exposure to agricultural chemicals play an important role in the development of cancer.

## Abbreviations

(NHL): Non-Hodgkin's Lymphoma; (ICD): International Classification of Diseases; (STS): Soft Tissue Sarcoma; (HL): Hodgkin lymphoma; (MM): Multiple Myeloma; (EBV): Epstein-Barr Virus; (SD): Standard deviation; (SE): Standard Error; (OR_adj_): Adjusted Odds Ratio; (95% CI): 95% confidence Intervel; (IARC): International Agency for Research on Cancer.

## Competing interests

The authors declare that they have no competing interests.

## Authors' contributions

HHM's main contribution was grant writing, study design, questionnaires development, and study coordination. The concept of including family history variables in this study was HHM's idea and she prepared the manuscript. PP was involved at the grant writing stage; she participated in the design of the study, and in the development of the questionnaires. PP was the national coordinator and biostatistician for this study. PP trained data managers from the six provinces about the data entry process, and supervised every stage of data cleaning. PP conducted the entire statistical analysis required for the manuscript and contributed to the preparation of the manuscript in sections relevant to statistical analysis. CPK made contributions to the statistical analysis and manuscript preparation. JJS was the co-investigator and British Columbia representative who participated in the design of the study and who supervised any data collection for the province of British Columbia. JAD and HHM were co-principal investigators of the study. JAD's main contribution was grant writing and questionnaires development. All authors read and approved the final manuscript.

## Pre-publication history

The pre-publication history for this paper can be accessed here:

http://www.biomedcentral.com/1471-2407/9/70/prepub

## Supplementary Material

Additional file 1 **Table 1.** Age distribution of cases and controls and descriptive Characteristics of the Families Studied: Numbers of first degree relatives* stratified by relationship to the index subjects**. This is a table of age distribution of cases and controls and descriptive characteristics of first degree relatives. Click here for file

Additional file 2 **Table 2.** Descriptive characteristics of the families studied: family size*** categorized as small, medium, large and largest excluding the index subjects. This is a table of descriptive characteristics of family size of the families studied. Click here for file

Additional file 3 **Table 3.** Descriptive characteristics of the families studied: distribution of number of reported generations with at least one family member affected with cancer excluding the index subjects. This is a table of the distribution of number of reported generations with at least one family member affected with cancer excluding the index subjects. Click here for file

Additional file 4 **Table 4.** Descriptive characteristics of the families studied: distribution of reported numbers**** of family members affected with cancer excluding the index subjects. This is a table of the distribution of reported number of family members affected with cancer excluding the index subjects. Click here for file

Additional file 5 **Table 5: Cancer in family relatives stratified by age distribution.** This is a table of the distribution of age of family relatives who are with cancer. Click here for file

Additional file 6 **Table 6.** Comparisons of families of cases of HL, MM, NHL, STS to families of controls: relationship of relatives with cancer to index subjects. This is a table of comparisons of families of cases of HL, MM, NHL, STS to families of controls. Click here for file

Additional file 7 **Table 7.** Characteristics of index subjects among those with and without a family history of cancer. This is a table of characteristics of index subjects among those with and without a family history of cancer. Click here for file

## References

[B1] Strong LC Genetic Etiology of Cancer Cancer 1977 40 438 444 10.1002/1097-0142(197707)40:1+<438::AID-CNCR2820400705>3.0.CO;2-I 328131

[B2] Mulvihill JJ Tulinius H Cancer Ecogenetics: Studying Genetic and Environment Interactions through Epidemiology Int J Epidemiol 1987 16 3 337 340 10.1093/ije/16.3.337 3667028

[B3] Khoury MJ Beaty TH Liang K Can Familial Aggregation of Disease Be Explained by Familial Aggregation of Environmental Risk Factors? Am J Epidemiol 1988 127 3 674 683 3341366 10.1093/oxfordjournals.aje.a114842

[B4] Wong O Raabe GK Non-Hodgkin's lymphoma and exposure to benzene in a multinational cohort of more than 308,000 petroleum workers, 1937 to 1996 J Occup Environ Med 2000 42 554 568 10.1097/00043764-200005000-00016 10824308

[B5] Smith MT Jones RM Smith AH Benzene exposure and risk of non-Hodgkin lymphoma Cancer Epidemiol Biomarkers Prev 2007 16 385 391 10.1158/1055-9965.EPI-06-1057 17337645

[B6] Merthi M Raynal H Cahuzac E Vinson F Cravedi JP Gamet-Payrastre L Occupational exposure to pesticides and risk of hematopoietic cancers: meta-analysis of case-control studies Cancer Causes Control 2007 18 10 1209 1226 10.1007/s10552-007-9061-1 17874193

[B7] Orsi L Troussard X Monnereau A Berthou C Fenaux P Marit G Soubeyran P Huguet F Milpied N Leporrier M Hemon D Clavel J Occupation and lymphoid malignancies: results from a French case-control study J Occup Environ Med 2007 49 12 1339 1350 18231081 10.1097/JOM.0b013e3181569a49

[B8] Mahajan R Blair A Coble J Lynch CF Hoppin JA Sandler DP Alavanja MC Carbaryl exposure and incident cancer in the Agricultural Health Study Int J Cancer 2007 121 8 1799 1805 10.1002/ijc.22836 17534892

[B9] Purdue MP Hoppin JA Blair A Dosemeci M Alavanja MC Occupational exposure to organochlorine insecticides and cancer incidence in the Agricultural Health Study Int J Cancer 2007 120 3 642 649 17096337 10.1002/ijc.22258 PMC1971137

[B10] Dreiher J Kordysh E Non-Hodgkin lymphoma and pesticide exposure: 25 years of research Acta Haematol 2006 116 3 153 164 10.1159/000094675 17016033

[B11] Miligi L Costantini AS Benvenuti A Kriebel D Bolejack V Tumino R Ramazzotti V Rodella S Stagnaro E Crosignani P Amadori D Mirabelli D Sommani L Belletti I Troschel L Romeo L Miceli G Tozzi GA Mendico I Vineis P Occupational exposure to solvents and the risk of lymphomas Epidemiology 2006 17 5 552 561 10.1097/01.ede.0000231279.30988.4d 16878041

[B12] Pahwa P Mcduffie HH Dosman JA McLaughlin JR Spinelli JJ Robson D Fincham S Hodgkin lymphoma, multiple myeloma, soft tissue sarcomas, insect repellents, and phenoxyherbicides J Occup Environ Med 2006 48 3 264 274 10.1097/01.jom.0000183539.20100.06 16531830

[B13] Fritschi L Benke G Hughes AM Kricker A Vajdic CM Grulich A Turner J Milliken S Kaldor J Armstrong BK Risk of non-Hodgkin lymphoma associated with occupational exposure to solvents, metals, organic dusts and PCBs (Australia) Cancer Causes control 2005 16 5 599 607 10.1007/s10552-004-7845-0 15986116

[B14] Zheng T Zahm SH Cantor KP Weisenburger DD Zhang Y Blair A Agricultural exposure to carbamate pesticides and risk of non-Hodgkin lymphoma J Occup Environ Med 2001 43 7 641 649 10.1097/00043764-200107000-00012 11464396

[B15] Persson B Occupational exposure and malignant lymphoma Int J Occup Med Environ Health 1996 9 4 309 321 9117190

[B16] Wiggle DT Semenciw RM Wilkins K Riedel D Ritter L Morrison H Mao Y Mortality Study of Canadian Farm Operators: non-Hodgkin's Lymphoma Mortality and agricultural Practices in Saskatchewan J Natl Cancer Inst 1990 82 575 580 10.1093/jnci/82.7.575 2313734

[B17] Gallagher RP Spinelli J Elwood JM Skippen EH Allergies and Agricultural Exposure as Risk Factors for Multiple Myeloma Br J Cancer 1983 48 853 857 6652026 10.1038/bjc.1983.277 PMC2011571

[B18] Hoar SK Blair A Holmes F Boysen C Robel RJ Hoover R Fraumeni JF Agricultural Herbicide Use and Risk of Lymphoma and Soft Tissue Sarcoma J Am Med Assn 1986 256 1141 1147 10.1001/jama.256.9.1141 3801091

[B19] Woods JS Polissar L Severson RK Heuser LS Kulander EG Soft Tissue Sarcoma and non-Hodgkin's Lymphoma in Relation to Phenoxyherbicide and Chlorinated Phenol Exposure J Natl Cancer Inst 1987 78 899 910 3471999

[B20] Morris PD Koepsell TD Daling JR Taylor JW Lyon JL Swanson GM Child M Weiss NS Toxic Substance Exposure and Multiple Myeloma: A Case-control Study J Natl Cancer Inst 1986 76 987 994 3458965

[B21] Hoar-Zahm S Weisenberger DD Babbit PA Saul RC Vaught JB Cantor KP Blair A A Case-control Study of non-Hodgkin's Lymphoma and the Herbicide 2,4-dichlophenoxyacetic Acetic Acid in Eastern Nebraska Epidemiology 1990 1 5 349 356 2078610 10.1097/00001648-199009000-00004

[B22] Wingren G Fredrikson M Brage HN Nordenskjold B Axelson O Soft Tissue Sarcoma and Occupational Exposures Cancer 1990 66 806 811 10.1002/1097-0142(19900815)66:4<806::AID-CNCR2820660435>3.0.CO;2-U 2386907

[B23] Hansen ES Lander F Lauritsen JM Time trends in cancer risk and pesticide exposure, a long-term follow-up of Danish gardeners Scand J Work Environ Health 2007 33 6 465 469 18327515 10.5271/sjweh.1162

[B24] Briggs NC Levine RS Hall HI Cosby O Brann EA Hennekens CH Occupational risk factors for selected cancers among African American and White men in the United States Am J Public Health 2003 93 10 1748 1752 14534232 10.2105/AJPH.93.10.1748 PMC1448044

[B25] Hoppin JA Tolbert PE Flanders WD Zhang RH Daniels DS Ragsdale BD Brann EA Occupational risk factors for sarcoma subtypes Epidemiology 1999 10 3 300 306 10.1097/00001648-199905000-00019 10230842

[B26] Hoppin JA Tolbert PE Herrick RF Freedman DS Ragsdale BD Horvat KR Brann EA Occupational chlorophenol exposure and soft tissue sarcoma risk among men aged 30–60 years Am J Epidemiol 1998 148 7 693 703 9778176 10.1093/aje/148.7.693

[B27] Kirkeleit J Riise T Bratveit M Moen BE Increased risk of acute myelogenous leukemia and multiple myeloma in a historical cohort of upstream petroleum workers exposed to crude oil Cancer Causes Control 2008 19 1 13 23 10.1007/s10552-007-9065-x 17906934

[B28] Cuzik J De Stavola B Multiple Myeloma, a Case-control Study Br J Cancer 1988 57 516 520 3395559 10.1038/bjc.1988.118 PMC2246387

[B29] Zheng T Zahm SH Cantor KP Weisenburger DD Zhang Y Blair A Agricultural Exposure to Carbamate Pesticides and Risk of Non-Hodgkin Lymphoma J Occup Environ Med 2001 43 641 649 10.1097/00043764-200107000-00012 11464396

[B30] Zahm SH Weisenburger DD Saal RC Vaught JB Babbit PA Blair A Pesticide Use, Genetic Susceptibility, and Non-Hodgkin's Lymphoma in Women 1994 Agricultural Health and Safety: Workplace, Environment, Sustainability, CRC Lewis Publishers, Boca Raton, USA 127 133

[B31] Cartwright RA Watkins G Epidemiology of Hodgkin's disease: a review Hematol Oncol 2004 22 1 11 26 10.1002/hon.723 15152367

[B32] Chiu BC Weisenburger DD Zahm SH Cantor KP Gapstur SM Holmes F Burmeister LF Blair A Agricultural pesticide use, familial cancer, and risk of non-Hodgkin lymphoma Cancer Epidemiol Biomarkers Prev 2004 13 4 525 531 15066915

[B33] Pahwa P McDuffie HH Dosman JA Robson D McLaughlin JR Spinelli JJ Fincham S Exposure to animals and selected risk factors among Canadian farm residents with Hodgkin's disease, multiple myeloma, or soft tissue sarcoma J Occup Environ Med 2003 45 8 857 868 10.1097/01.jom.0000083033.56116.c1 12915787

[B34] Altieri A Bermejo JL Hemminki K Familial risk for non-Hodgkin lymphoma and other lymphoproliferative malignancies by histopathologic subtype: the Swedish Family-Cancer Database Blood 2005 106 668 672 10.1182/blood-2005-01-0140 15811955

[B35] Blair A Linos A Stewart PA Burmeister LF Gibson R Everett G Schuman L Cantor KP Comments on Occupational and Environmental Factors in the Origen of Non-Hodgkin's Lymphoma Cancer Res 1992 52 5501s 5502s 1394163

[B36] Pearce N Bethwaite P Increasing Incidence of Non-Hodgkin's Lymphoma: Occupational and Environmental Factors Cancer Res 1992 52 5496s 5500s 1394162

[B37] Eriksson M Karlsson M Occupational and Other Environmental Factors and Multiple Myeloma: A Population Based Case-control Study Br J Ind Med 1992 49 95 103 1536825 10.1136/oem.49.2.95 PMC1012073

[B38] McDuffie HH Pahwa P McLaughlin JR Spinelli JJ Fincham S Dosman JA Robson D Skinnider LF Choi NW Non-Hodgkin's Lymphoma and Specific Pesticide Exposures in Men: Cross-Canada Study of Pesticides and Health Cancer Epidemiol Biomarkers Prev 2001 10 1155 1163 11700263

[B39] Linet MS Pottern LM Familial Aggregation of Hematopoietic Malignancies and Risk of Non-Hodgkin's Lymphoma Cancer Res (suppl) 1992 52 5465s 5473s 1394155

[B40] Goldgar DE Easton DF Cannon-Allright LA Skolnick MH Systematic Population-based Assessment of Cancer Risk in First Degree Relatives of Cancer Probands J Natl Cancer Inst 1994 86 1600 1608 10.1093/jnci/86.21.1600 7932824

[B41] Bourguet CC Grufferman S Delzell E deLong ER Cohen HJ Multiple Myeloma and Family History of Cancer. A Case-control Study Cancer 1985 56 2133 2139 10.1002/1097-0142(19851015)56:8<2133::AID-CNCR2820560842>3.0.CO;2-F 4027940

[B42] Lichtenstein P Holm NV Verkasalo PK Iliadou A Kaprio J Koskenvuo M Pukkala E Skytthe A Hemminki K Environmental and Heritable Factors in the Causation of Cancer N Engl J Med 2000 343 78 85 10.1056/NEJM200007133430201 10891514

[B43] Haim N Cohen Y Robinson E Malignant Lymphoma in First-degree Blood Relatives Cancer 1982 49 2197 2200 10.1002/1097-0142(19820515)49:10<2197::AID-CNCR2820491036>3.0.CO;2-9 7074535

[B44] Moutou C Le Bihan C Chompret Agnes Poisson N Brugieres L Bressac Feunteun J Lemerle J Bonaiti-Pellie C Genetic Transmission of Susceptibility to Cancer in Families of Children with Soft Tissue Sarcomas Cancer 1996 78 1483 1491 10.1002/(SICI)1097-0142(19961001)78:7<1483::AID-CNCR16>3.0.CO;2-W 8839555

[B45] Brown LM Linet MS Greenberg RS Silverman DT Hayes RB Swanson GM Schwartz AG Schoenberg JB Pottern LM Fraumeni JF Jr Multiple Myeloma and Family History of Cancer among Blacks and Whites in the U.S Cancer 1999 85 2385 2390 10.1002/(SICI)1097-0142(19990601)85:11<2385::AID-CNCR13>3.0.CO;2-A 10357409

[B46] Mack TM Cozen W Shibata DK Weiss LM Nathwani BN Hernandez AM Taylor CR Hamilton AS Deapen DM Rappaport EB Concordance for Hodgkin's Disease in Identical Twins Suggesting Genetic Susceptibility to the Young-adult Form of the Disease N Engl J Med 1995 332 413 418 10.1056/NEJM199502163320701 7824015

[B47] Le Bihan C Moutou C Chompret A Abel A Poisson N Brugieres L Lemerle J Bonaiti-Pellie C Cancers in relatives of children with non-Hodgkin's lymphoma Leuk Res 1996 20 181 186 10.1016/0145-2126(95)00138-7 8628018

[B48] Hemminski K Vaittinen P Kyyronen P Age-specific Familial Risks in Common Cancers of the Offspring Int J Cancer 1998 78 172 175 10.1002/(SICI)1097-0215(19981005)78:2<172::AID-IJC9>3.0.CO;2-W 9754648

[B49] Verkasalo PK Kaprio J Koskenvuo M Pukkala E Genetic Predisposition, Environment and Cancer Incidence: A Nationwide Twin study in Finland, 1976–1995 Int J Cancer 1999 83 743 749 10.1002/(SICI)1097-0215(19991210)83:6<743::AID-IJC8>3.0.CO;2-Q 10597189

[B50] Zhu K Levine RS Brann EA Gu Y Caplan LS Hall I Baum MK Risk Factors for non-Hodgkin's Lymphoma According to Family History of Haematolymphoproliferative Maligancies Int J Epidemiol 2001 30 818 824 10.1093/ije/30.4.818 11511610

[B51] Bondy ML Lustbader ED Strom SS Strong LC Segregation Analysis of 159 Soft Tissue Sarcoma Kindreds: Comparison of Fixed and Sequential sampling Schemes Genetic Epidemiol 1992 9 291 304 10.1002/gepi.1370090502 1427019

[B52] Hemminki K Vaittinen P National Database of Familial Cancer in Sweden Genetic Epidemiol 1998 15 225 236 10.1002/(SICI)1098-2272(1998)15:3<225::AID-GEPI2>3.0.CO;2-3 9593110

[B53] Ogawa H Kato I Tominaga S Family History of Cancer among Cancer Patients Jpn J Cancer Res 1985 76 113 118 3920100

[B54] Goldin LR Pfeiffer RM Gridley G Gail MH Li X Mellemkjaer L Olsen JH Hemminki K Linet MS Familial aggregation of Hodgkin lymphoma and related tumors Cancer 2004 100 1902 1908 10.1002/cncr.20189 15112271

[B55] Berstein R Ross RK Prior Medication Use and Health History as Risk Factors for non-Hodgkin's Lymphoma: Preliminary results from a Case-control Study in Los Angeles County Cancer Res (suppl) 1992 52 5510s 5515s 1394165

[B56] Vena JE Bona JR Byers TE Middleton E Swanson MK Graham S Allergy-related Diseases and Cancer: An Inverse Association Am J Epidemiol 1985 122 66 74 4014202 10.1093/oxfordjournals.aje.a114087

[B57] Briggs NC Levine RS Brann EA Allergies and Risk of Non-Hodgkin's Lymphoma by Subtype Cancer Epidemiol Biomarkers Prev 2002 11 401 407 11927501

[B58] Herrinton LJ Friedman GD Cigarette Smoking and Risk of Non-Hodgkin's Lymphoma Subtypes Cancer Epidemiol Biomarkers Prev 1998 7 25 28 9456239

[B59] Brown LM Everett GD Gibson R Burmeister LF Schuman LM Blair A Smoking and Risk of non-Hodgkin's Lymphoma and Multiple Myeloma Cancer Causes Control 1992 3 49 55 10.1007/BF00051912 1536913

[B60] Linet MS McLaughlin JK Hsing AW Wacholder S Co Chien HT Schuman LM Bjelke E Blot WJ Is Cigarette Smoking a Risk Factor for Non-Hodgkin's Lymphoma or Multiple Myeloma? Leuk Res 1992 16 621 624 10.1016/0145-2126(92)90011-U 1635380

[B61] Doody MM Linet MS Glass AG Curtis RE Pottern LM Rush BB Boice JD Jr Fraumeni JF Jr Friedman GD Risks of Non-Hodgkin's Lymphoma, Multiple Myeloma, and Leukemia Associated with Common Medications Epidemiol 1996 7 131 139 10.1097/00001648-199603000-00005 8834551

[B62] Linet MS McLaughlin JK Harlow SD Fraumeni JF Family History of Autoimmune Disorders and Cancer in Multiple Myeloma Int J Epidemiol 1988 17 512 513 10.1093/ije/17.3.512 3209328

[B63] Lynch HT Marcus JN Weisenburger DD Watson P Fitzimmons ML Grierson H Smith DM Lynch J Purtilo D Genetic and Immunopathological Findings in a Lymphoma Family Br J Cancer 1989 59 622 626 2713249 10.1038/bjc.1989.126 PMC2247145

[B64] Cancer and the Environment National Institute of Health NIH Publication No. 03-2039 http://www.niehs.nih.gov/health/scied/documents/CancerEnvironment.pdf Available from (Access on Januray 5, 2009)

[B65] The Environment, Cancer and You Canadian Cancer Society 2008 http://www.cancer.ca/Canada-wide/Prevention/Cancer and the environment.aspx?sc_lang=en Available from (Access on Januray 5, 2009)

[B66] McDuffie HH Clustering of Cancer in Families of Patients with Primary Lung Cancer J Clin Epidemiol 1991 44 69 76 10.1016/0895-4356(91)90202-K 1986060

[B67] SPSS-Data Entry II Statistical Package for the Social Sciences Statistical Data Analysis 1998 SPSS Inc. Chicago Illinois

[B68] Breslow NE Day NE The Analysis of Case-control Studies Statistical Methods in Cancer Research 1980 1 IARC Sci Publ.32 Lyon, France 7216345

[B69] SAS Institute The SAS System for Windows, Release 9.03 2007 SAS Institute, Cary, NC. USA

[B70] Mulvihill JJ Clinical Ecogenetics. Cancer in Families Editorial, N Engl J Med 1985 312 1569 1570 10.1056/NEJM198506133122410 4000188

[B71] Bondy ML Strom SS Colopy MW Brown BW Strong LC Accuracy of family history of cancer obtained through interviews with relatives of patients with childhood sarcoma J Clin Epidemiol 1994 47 89 96 10.1016/0895-4356(94)90037-X 8283198

[B72] Albert S Child M Familial cancer in the general populations Cancer 1977 40 1674 1679 10.1002/1097-0142(197710)40:4<1674::AID-CNCR2820400442>3.0.CO;2-C 907977

[B73] Albano WA Lynch HT Recabaren JA Organ CH Mailliard JA Black LE Follett KL Lynch J Familial cancer in an oncology clinic Cancer 1981 47 2113 2118 10.1002/1097-0142(19810501)47:9<2113::AID-CNCR2820470902>3.0.CO;2-S 7226103

[B74] Floderus B Barlow L Mack TM Recall bias in subjective reports of familial cancer Epidemiol 1990 1 318 321 10.1097/00001648-199007000-00011 2083311

[B75] Love RR Evans AM Josten DM The accuracy of patient reports of a family history of cancer J Chronic Dis 1985 38 289 293 10.1016/0021-9681(85)90074-8 3998045

[B76] Ottman R An epidemiologic approach to gene-environment interaction Genetic Epidemiol 1990 7 177 185 10.1002/gepi.1370070302 PMC2819318 2369997

[B77] Mills PK Newell GR Beeson WL Fraser GE Phillips RL History of cigarette smoking and risk of leukemia and myeloma: Results from the Adventist Health Study J Natl Cancer Inst 1990 82 1832 1836 10.1093/jnci/82.23.1832 2250299

[B78] Linet MS Pottern LM Familial aggregation of hematopoietic malignancies and risk of non-Hodgkin's lymphoma Cancer Res (supp) 1992 52 5468s 5473s 1394155

[B79] Novakovic B Goldstein AM Tucker MA Validation of family history of cancer in deceased family members J Natl Cancer Inst 1996 88 1492 1493 10.1093/jnci/88.20.1492 8841026

[B80] Schneider NR Chaganti SR German J Chaganti SK Familial predisposition to cancer and age at onset of disease in randomly selected cancer patients Am J Hum Genet 1983 35 454 467 6859041 PMC1685650

