# Supplementary material for: Clustering of cancer among families of cases with Hodgkin Lymphoma (HL), Multiple Myeloma (MM), Non-Hodgkin's Lymphoma (NHL), Soft Tissue Sarcoma (STS) and control subjects
Source: BMC Cancer. 2009 Feb 27;9:70. doi: 10.1186/1471-2407-9-70 (PMC2653543; doi:10.1186/1471-2407-9-70)
Supplement: Additional file 1 — Table 1. Age distribution of cases and controls and descriptive Characteristics of the Families Studied: Numbers of first degree relatives* stratified by relationship to the index subjects**. This is a table of age distribution of cases and controls and descriptive characteristics of first degree relatives. [file 1471-2407-9-70-S1.pdf]

Table 1. Age distribution of cases and controls and descriptive Characteristics of the Families Studied: Numbers of first degree relatives* stratified by relationship to the index subjects**

	HL (n=316)		MM (n=342)		NHL (n=513)		STS (n=357)		Control (n=1506)	
Age distribution, in years	n	%	n	%	n	%	n	%	n	%
≤ 40	183	57.9	9	2.6	63	12.3	88	24.6	356	23.6
>40, ≤49	56	17.7	24	7.0	78	15.2	49	13.7	221	14.7
> 49, ≤59	28	8.9	59	17.3	111	21.6	54	14.8	248	16.5
>59, ≤69	26	8.2	116	33.9	137	26.7	71	19.9	362	24.0
> 69	23	7.3	134	39.2	124	24.2	96	26.9	319	21.2
Mean ± SD	40.2 ± 0.90		64.7 ± 0.60		57.7 ± 0.63		54.7 ± 0.90		54.1 ± 0.42	
	n	mean ± SD	n	mean ± SD	n	mean ± SD	n	mean ± SD	n	mean ± SD
Siblings	946	3.08 ± 2.6	1390	4.84 ± 3.2	2070	4.88 ± 3.3	1313	4.48 ± 3.0	5662	4.55 ± 3.1
Brothers	505	1.91 ± 1.6	722	2.42 ± 1.9	1080	2.46 ± 2.0	660	2.18 ± 1.7	2986	2.30 ± 1.9
Sisters	441	1.65 ± 1.3	668	2.29 ± 1.8	990	2.24 ± 1.8	653	2.14 ± 1.7	2676	2.09 ± 1.6
Children	424		983		1301		796		3385	
Sons	224	0.86 ± .95	518	1.77 ± 1.2	661	1.53 ± 1.5	414	1.39 ± 1.3	1788	1.42 ± 1.2
Daughters	200	0.79 ± 1.0	465	1.60 ± 1.3	640	1.51 ± 1.4	382	1.36 ± 1.4	1597	1.31 ± 1.2
Mean first degree family members including parents	6.33		8.94		8.57		7.93		8.01	

* first degree relatives include parents, siblings and offspring of the index subjects.
**index subjects were omitted
