# Supplementary material for: Clustering of cancer among families of cases with Hodgkin Lymphoma (HL), Multiple Myeloma (MM), Non-Hodgkin's Lymphoma (NHL), Soft Tissue Sarcoma (STS) and control subjects
Source: BMC Cancer. 2009 Feb 27;9:70. doi: 10.1186/1471-2407-9-70 (PMC2653543; doi:10.1186/1471-2407-9-70)
Supplement: Additional file 2 — Table 2. Descriptive characteristics of the families studied: family size*** categorized as small, medium, large and largest excluding the index subjects. This is a table of descriptive characteristics of family size of the families studied. [file 1471-2407-9-70-S2.pdf]

Table 2. Descriptive Characteristics of the Families Studied: Family size*** categorized as small, medium, large and largest excluding the index subjects.

	HL		MM		NHL		STS		Controls	
Family size	n	% of families	n	% of families	n	% of families	n	% of families	n	% of families
Small	187	59.2	140	40.9	227	44.2	177	49.6	735	48.8
Medium	64	20.2	58	17.0	83	16.2	60	16.8	219	14.5
Large	36	11.4	51	14.9	82	16.0	60	16.8	271	18.0
Largest	29	9.2	93	27.2	121	23.6	60	16.8	281	18.7

Chi-square = 61.5, df = 12, p < .0001

Separate Chi-squared values: for HL χ^2=31.57, df=3, p<0.0001; for MM χ^2=16.28, df=3, p<0.001; for NHL χ^2=7.89, df=3, p<0.05; for STS χ^2=1.80, df=3, p>0.05.

*** Small: (<= 2 siblings/offspring plus parents); Medium: (>= 3 and <= 5 siblings/offspring plus parents) Large: (>= 6 and <= 8 siblings/offspring plus parents); Largest: (>=9siblings/ offspring plus parents)
