# Supplementary material for: Clustering of cancer among families of cases with Hodgkin Lymphoma (HL), Multiple Myeloma (MM), Non-Hodgkin's Lymphoma (NHL), Soft Tissue Sarcoma (STS) and control subjects
Source: BMC Cancer. 2009 Feb 27;9:70. doi: 10.1186/1471-2407-9-70 (PMC2653543; doi:10.1186/1471-2407-9-70)
Supplement: Additional file 3 — Table 3. Descriptive characteristics of the families studied: distribution of number of reported generations with at least one family member affected with cancer excluding the index subjects. This is a table of the distribution of number of reported generations with at least one family member affected with cancer excluding the index subjects. [file 1471-2407-9-70-S3.pdf]

Table 3. Descriptive Characteristics of the Families Studied: Distribution of number of reported generations with at least one family member affected with cancer excluding the index subjects.

Generations Affected	HL		MM		NHL		STS		Controls	
	Number of families	OR adj[#] (95%CI)	Number of families	OR adj[j#] (95%CI)	Number of families	OR adj[#] (95%CI)	Number of families	OR adj[#] (95%CI)	Number of families	OR adj[#] (95%CI)
Zero	211	1	179	1	288	1	224	1	1012	Reference
One	96	**1.85(1.37,2.51)**	136	**1.37(1.05,1.79)**	178	1.34(1.07,1.68)	114	**1.29(1.00.1.68)**	422	
Two or more generations affected	9	1.93(0.89,4.17)	27	1.24(0.76,2.01)	47	**2.01(1.34,3.01)**	19	1.09(0.63,1.90)	72	

[#]All odds ratios were adjusted for age and province of residence
