# Supplementary material for: Clustering of cancer among families of cases with Hodgkin Lymphoma (HL), Multiple Myeloma (MM), Non-Hodgkin's Lymphoma (NHL), Soft Tissue Sarcoma (STS) and control subjects
Source: BMC Cancer. 2009 Feb 27;9:70. doi: 10.1186/1471-2407-9-70 (PMC2653543; doi:10.1186/1471-2407-9-70)
Supplement: Additional file 4 — Table 4. Descriptive characteristics of the families studied: distribution of reported numbers**** of family members affected with cancer excluding the index subjects. This is a table of the distribution of reported number of family members affected with cancer excluding the index subjects. [file 1471-2407-9-70-S4.pdf]

Table 4. Descriptive Characteristics of the Families Studied: Distribution of reported numbers**** of family members affected with cancer excluding the index subjects.

Number of Affected Family Members	HL		MM		NHL		STS		Controls	
	Number of families	% of families	Number of families	% of families	Number of families	% of families	Number of families	% of families	Number of families	% of families
Zero	211	66.8	179	52.3	288	56.1	224	62.7	1012	67.2
One	88	27.8	103	30.1	151	29.4	96	26.9	369	24.5
Two	13	4.1	34	9.9	52	10.1	25	7.0	86	5.7
Three	4	1.3	19	5.6	17	3.3	9	2.5	29	1.9
Four	0	0	5	1.5	5	1.0	1	0.3	8	0.5
Five or more	0	0	2	0.6	0		2	0.6	2	0.1
% Affected Families		33.2		47.7		43.9		37.8		32.8
% Two or More Affected Individuals/family		5.4		17.6		14.5		10.7		8.3

**** Chi-square = 59.8, df = 8, $p < .0001$ using the following categories: zero, one, ≥ 2 affected per family.
