# Supplementary material for: Clustering of cancer among families of cases with Hodgkin Lymphoma (HL), Multiple Myeloma (MM), Non-Hodgkin's Lymphoma (NHL), Soft Tissue Sarcoma (STS) and control subjects
Source: BMC Cancer. 2009 Feb 27;9:70. doi: 10.1186/1471-2407-9-70 (PMC2653543; doi:10.1186/1471-2407-9-70)
Supplement: Additional file 5 — Table 5: Cancer in family relatives stratified by age distribution. This is a table of the distribution of age of family relatives who are with cancer. [file 1471-2407-9-70-S5.pdf]

Table 5: Cancer in family relatives stratified by age distribution

Age distribution, in years	HL n (%)	MM n (%)	NHL n (%)	STS n (%)	Controls n (%)
At least one parent					
≤ 40	37 (20..3)	2 (22.2)	13 (20.6)	11 (12.5)	54 (15.2)
>40, ≤49	23 (41.1)	8 (33.3)	24 (30.8)	19 (38.8)	55 (24.9)
> 49, ≤59	10 (35.7)	21 (35.6)	33 (29.7)	13 (24.5)	72 (29.0)
>59, ≤69	6 (23.1)	28 (24.1)	44 (32.1)	22 (31.0)	102 (28.2)
> 69	5 (21.7)	27 (20.1)	39 (31.5)	25 (26.0)	79 (24.8)
At least one sibling					
≤ 40	7 (3.8)	1 (11.1)	5 (7.9)	3 (3.4)	3 (0.8)
>40, ≤49	5 (8.9)	3 (12.5)	8 (10.3)	3 (6.1)	9 (4.1)
> 49, ≤59	7 (25.0)	15 (25.4)	21 (18.9)	8 (15.1)	24 (9.7)
>59, ≤69	4 (15.4)	28 (24.1)	42 (30.7)	14 (19.7)	74 (20.4)
> 69	8 (34.8)	47 (35.1)	33 (26.6)	30 (31.2)	78 (24.5)
At least one child					
≤ 40	0 (0.0)	0 (0.0)	0 (0.0)	0 (0.0)	0 (0.0)
>40, ≤49	0 (0.0)	1 (4.2)	0 (0.0)	0 (0.0)	1 (0.5)
> 49, ≤59	1 (3.6)	2 (3.4)	4 (3.6)	0 (0.0)	1 (0.4)
>59, ≤69	0 (0.0)	0 (0.0)	2 (1.5)	2 (2.8)	6 (1.7)
> 69	1 (4.3)	8 (6.0)	4 (3.2)	2 (2.1)	10 (3.1)
