# Supplementary material for: Clustering of cancer among families of cases with Hodgkin Lymphoma (HL), Multiple Myeloma (MM), Non-Hodgkin's Lymphoma (NHL), Soft Tissue Sarcoma (STS) and control subjects
Source: BMC Cancer. 2009 Feb 27;9:70. doi: 10.1186/1471-2407-9-70 (PMC2653543; doi:10.1186/1471-2407-9-70)
Supplement: Additional file 6 — Table 6. Comparisons of families of cases of HL, MM, NHL, STS to families of controls: relationship of relatives with cancer to index subjects. This is a table of comparisons of families of cases of HL, MM, NHL, STS to families of controls. [file 1471-2407-9-70-S6.pdf]

Table 6. Comparisons of families of cases of HL, MM, NHL, STS to families of controls: relationship of relatives with cancer to index subjects.

	**HL**			**MM**			**NHL**			**STS**			**Controls**	
	n (%) of families		Adjusted OR[#] (95% CI)	n (%) of families		Adjusted OR[#] (95% CI)	n (%) of families		Adjusted OR[#] (95% CI)	n (%) of families		Adjusted OR[#] (95% CI)	n (%) of families	
Any first degree relative affected:														
Yes	104	(32.9)	**1.79 (1.33,2.42)**	164	(47.9)	**1.38 (1.07, 1.78)**	225	(43.9)	**1.43 (1.15, 1.77)**	133	(37.2)	**1.30 (1.00, 1.68)**	498	(33.1)
No	202	(63.9)		169	(49.4)		276	(53.8)		209	(58.5)		973	(64.6)
Unkonown/missing	10	(3.2)		9	(2.6)		12	(2.3)		15	4.2)		35	(2.3)
OR adjusted for family size			**2.02 (1.36, 3.01)**			1.32 (0.95, 1.84)			1.01 (0.98, 1.05)			1.14 (0.81, 1.60)		
Parents:														
1. Neither affected	235	(74.4)		256	(74.8)		360	(70.2)		267	(74.8)		1144	(76.0)
Mother affected, father unaffected	36	(11.4)	1.50 (0.98, 2.29)	33	(9.6)	0.91 (0.60, 1.39)	69	(13.4)	1.31 (0.95, 1.79)	38	(10.6)	1.14 (0.77, 1.68)	153	(10.2)
Father affected, mother unaffected	36	(11.4)	1.37 (0.91, 2.08)	37	(10.8)	0.79 (0.53, 1.17)	64	(12.5)	1.06 (0.77, 1.45)	42	(11.8)	1.10 (0.77, 1.59)	169	(11.2)
both parents affected	9	(2.8)	1.64 (0.74, 3.64)	16	(4.7)	1.53 (0.82, 2.83)	20	(3.9)	1.38 (0.79, 2.41)	10	(2.8)	0.99 (0.49, 2.02)	40	(2.6)
2. At least one affected parent	81	(25.6)	**1.57 (1.15, 2.14)**	86	(25.1)	0.91 (0.68, 1.22)	153	(29.8)	1.25 (0.99, 1.58)	90	(25.2)	1.12 (0.85, 1.48)	362	(24.0)
Siblings:														
1. At least one affected sibling	31	(9.8)	**1.95 (1.23, 3.08)**	94	(27.5)	**1.60 (1.19, 2.16)**	109	(21.2)	**1.66 (1.26, 2.18)**	58	(16.2)	1.30 (0.92, 1.84)	188	(12.5)
OR adjusted for total number of siblings			**1.94 (1.12, 3.34)**			**1.71 (1.22, 2.40)**			**1.68 (1.23, 2.31)**			1.35 (0.91, 2.00)		
2. At least one affected brother	15	(4.7)	1.53 (0.84, 2.79)	55	(16.1)	1.39 (0.97, 2.00)	63	(12.3)	**1.47 (1.05, 2.07)**	33	(9.2)	1.13 (0.74, 1.73)	114	(7.6)
OR adjusted for total number of brothers			1.74 (0.93, 3.25)			**1.47 (1.01, 2.13)**			**1.57 (1.10, 2.23)**			1.17 (0.75, 1.83)		
3. At least one affected sister	17	(5.4)	**1.89 (1.05, 3.38)**	54	(15.8)	**1.66 (1.14, 2.40)**	56	(10.9)	**1.52 (1.07, 2.17)**	32	(9.0)	1.34 (0.86, 2.08)	100	(6.6)
OR adjusted for total number of sisters			1.79 (0.97, 3.29)			**1.48 (1.01, 2.18)**			1.39 (0.96, 2.02)			1.24 (0.78, 1.96)		
Children:														
1. At least one affected child	2	(0.6)		11	(3.2)		10	(1.9)		4	(1.1)		18	(1.2)
1. At least one affected male	61	(19.3)	**1.70 (1.20, 2.41)**	101	(29.5)	1.18 (0.90, 1.56)	142	(27.7)	**1.38 (1.08, 1.76)**	78	(21.8)	1.11 (0.82, 1.49)	301	(20.0)
OR adjusted for total number of male relatives			**1.74 (1.17, 2.60)**			1.17 (0.85, 1.61)			**1.38 (1.04, 1.82)**			1.04 (0.73, 1.47)		
2. At least one affected female	59	(18.7)	**1.67 (1.18, 2.38)**	102	(29.8)	**1.46 (1.10, 1.94)**	127	(24.8)	**1.31 (1.02, 1.67)**	76	(21.3)	1.20 (0.89, 1.62)	282	(18.7)
OR adjusted for total number of female relatives			**1.82 (1.21, 2.76)**			**1.50 (1.08, 2.07)**			1.14 (0.85, 1.53)			1.05 (0.73, 1.51)		

Families ascertained through control subjects constituted the reference group. Index subjects were omitted from these analyses.

[#]All odds ratios were adjusted for age and province of residence
