# Supplementary material for: Clustering of cancer among families of cases with Hodgkin Lymphoma (HL), Multiple Myeloma (MM), Non-Hodgkin's Lymphoma (NHL), Soft Tissue Sarcoma (STS) and control subjects
Source: BMC Cancer. 2009 Feb 27;9:70. doi: 10.1186/1471-2407-9-70 (PMC2653543; doi:10.1186/1471-2407-9-70)
Supplement: Additional file 7 — Table 7. Characteristics of index subjects among those with and without a family history of cancer. This is a table of characteristics of index subjects among those with and without a family history of cancer. [file 1471-2407-9-70-S7.pdf]

Table 7. Characteristics of index subjects among those with and without a family history of cancer

	HL		Age/Province Adjusted OR (95% CI)	MM		Age/Province Adjusted OR (95% CI)	NHL		Age/Province Adjusted OR (95% CI)	STS		Age/Province Adjusted OR (95% CI)	Controls	
	n	%		n	%		n	%		n	%		n	%
**Pesticide Exposure and Family History of Cancer**														
Pesticide Exposure(-) and Family History of Cancer(-)	155	49.1	reference	145	42.4	reference	215	41.9	reference	172	48.2	reference	778	51.7
Pesticide Exposure (+) and Family History of Cancer (-)	56	17.7	1.12 (0.77, 1.64)	34	9.9	0.85 (0.56, 1.29)	73	14.2	1.23 (0.89, 1.68)	52	14.6	1.00 (0.70,1.43)	234	15.5
Pesticide Exposure(-) and Family History of Cancer(+)	85	26.9	**2.25 (1.61, 3.15)**	112	32.8	1.18 (0.88, 1.58)	161	31.4	**1.43 (1.12, 1.83)**	95	26.6	1.25 (0.93, 1.68)	364	24.2
Pesticide Exposure (+) and Family History of Cancer(+)	20	6.3	1.17 (0.67, 2.01)	51	14.9	**1.69 (1.14, 2.51)**	64	12.5	**1.72 (1.21, 2.45)**	38	10.6	1.30 (0.86,1.98)	130	8.6
**Personal Smoking History and Family History of Cancer**														
Nonsmoker and Family History of cancer(-)	98	31.6	reference	44	13.1	reference	95	19.0	reference	91	26.3	reference	383	26.0
Current or exsmoker and Family History of Cancer(-)	108	34.8	1.20 (0.85, 1.70)	132	39.2	**1.46 (1.01, 2.12)**	185	36.9	1.04 (0.79, 1.38)	125	36.1	0.85 (0.63, 1.15)	606	41.2
Nonsmoker and Family History of Cancer(+)	30	9.7	**1.68 (1.01, 2.78)**	43	12.8	**1.73 (1.07, 2.80)**	65	13.0	1.53 (1.05, 2.22)	40	11.6	1.11 (0.72, 1.71)	143	9.7
Current or exsmoker and Family History of Cancer(+)	4	23.9	**2.34 (1.57, 3.48)**	118	35.0	**1.79 (1.22, 2.63)**	156	31.1	**1.45 (1.07, 1.96)**	90	26.0	1.14 (0.81, 1.61)	340	23.1
**Age at diagnosis (recruitment for controls)***	Mean ± SD			Mean ± SD			Mean ± SD			Mean ± SD			Mean ± SD	
Family history of Cancer (-)	37.7 ± 15			64.3 ± 11			55.8 ± 15			51.8 ± 18			51.6 ± 17	
Family history of Cancer (+)	45.0 ± 15			65.1 ± 11			60.3 ± 13			59.9 ± 15			59.3 ± 14	
T-test (p)	-3.95 **(<0.0001)**			- 0.71 (0.48)			- 3.71 **(0.0002)**			- 4.78 **(< 0.0001)**			- 9.35 (< **0.0001)**	

* All these comparisons are within the case or control category.
